# Cell types and synchronous-activity patterns of inspiratory neurons in the preBötzinger complex of mouse medullary slices during early postnatal development

**DOI:** 10.1038/s41598-023-27893-w

**Published:** 2023-01-11

**Authors:** Yoshihiko Oke, Fumikazu Miwakeichi, Yoshitaka Oku, Johannes Hirrlinger, Swen Hülsmann

**Affiliations:** 1 Division of Physiome, Department of Physiology, Hyogo Medical University, 1-1 Mukogawa-cho, Nishinomiya, 663-8501 Japan; 2 Department of Statistical Modeling, The Institute of Statistical Mathematics, 10-3, Midori-cho, Tachikawa, 190-0014 Japan; 3 Department of Statistical Science, School of Multidisciplinary Sciences, The Graduate University for Advanced Studies, Shounan-Kokusai-Mura, Hayama-cho, Miura, 240-0193 Japan; 4 Carl-Ludwig-Institute for Physiology, Faculty of Medicine, University of Leipzig, Liebigstraße 27, 04103 Leipzig, Germany; 5 Department of Neurogenetics, Max Planck Institute for Multidisciplinary Science, Hermann-Rein-Straße 3, 37075 Göttingen, Germany; 6 Department of Anesthesiology, University Medical Center, Humboldtallee 23, 37073 Göttingen, Germany

**Keywords:** Respiration, Cellular neuroscience, Development of the nervous system, Neural circuits

## Abstract

To examine whether and how the inspiratory neuronal network in the preBötzinger complex (preBötC) develops during the early postnatal period, we quantified the composition of the population of inspiratory neurons between postnatal day 1 (p1) and p10 by applying calcium imaging to medullary transverse slices in double-transgenic mice expressing fluorescent marker proteins. We found that putative excitatory and glycinergic neurons formed a majority of the population of inspiratory neurons, and the composition rates of these two inspiratory neurons inverted at p5–6. We also found that the activity patterns of these two types of inspiratory neurons became significantly well-synchronized with the inspiratory rhythmic bursting pattern in the preBötC within the first postnatal week. GABAergic and GABA-glycine cotransmitting inspiratory neurons formed only a small population just after birth, which almost disappeared until p10. In conclusion, the inspiratory neuronal network in the preBötC matures at the level of both neuronal population and neuronal activities during early postnatal development.

## Introduction

The preBötzinger complex (preBötC), one of the respiration centres in the ventrolateral medulla, generates spontaneous inspiratory rhythmic bursts even in an isolated transverse slice^1,2^. In the preBötC of medullary slice preparations, inspiratory rhythmic activity emerges before birth, at embryonic day 15 (E15) in mice and E16.5–17 in rats^3–5^. The neuronal network for the generation of the inspiratory rhythm in the preBötC is considered to develop after the onset of rhythmic activity. In the preBötC in slice preparations from rats, the inspiratory rhythm frequency and the amplitude of inspiratory motor discharge increase from E17 to postnatal day 2 (p2), while burst duration and the coefficient of variation of the burst interval decrease^4^. Additionally, in vivo, the respiratory frequency increases in neonatal mice between p0 and p12^6,7^, and the variability of the respiratory cycle interval decreases after p3^6^. Therefore, the inspiratory neuronal network in the preBötC appears to further develop after birth. However, it remains unexamined whether and how the inspiratory neuronal network structure in the preBötC develops at the postnatal stage.

The composition of the population of inhibitory neurons in the preBötC changes during the first 2 postnatal weeks^8^. Using double transgenic (TG) mice expressing EGFP in the neuronal glycine transporter-positive (GlyT2^+^) neurons and tdTomato in 65-kD isoform of glutamic acid decarboxylase-positive (GAD65^+^) neurons, three types of inhibitory neurons were identified in the preBötC by gene/fluorescent protein expression pattern: glycinergic (GlyT2^+^/GAD65^-^), GABAergic (GlyT2^-^/GAD65^+^), and presumed GABA-glycine cotransmitting neurons (GGCN: GlyT2^+^/GAD65^+^). During late embryonic development, most inhibitory neurons in the preBötC are GGCN. The GGCN starts to differentiate into GABAergic neurons around birth and into glycinergic neurons at approximately p4. This differentiation of GGCN is almost completed by approximately p15. Meanwhile, the preBötC contains numerous noninspiratory neurons (> 50% of GlyT2^+^ neurons and > 80% of GAD67^+^ neurons) as well as inspiratory neurons^9–12^. In addition, inhibitory neurotransmitters change their effect on a subset of postsynaptic neurons during the early postnatal period. For example, the reversal potential of the GABA_A_-receptor-activated membrane response (E_GABA-A_) progressively decreases from −14 mV at p0 to −72 mV at p4 in the preBötC of mouse slices^11^. Consistently, inhibition of GABA_A_ receptors and glycine receptors increases the rhythmic burst frequency in the hypoglossal motor nucleus in p7–15 mouse rhythmic slice preparations but has no effect on the burst frequency at p0–3^13^. Thus, the inspiratory neuronal network might develop during the early postnatal period, but the details remain unknown.

In the present study, we hypothesized that the neuronal network in the preBötC develops during the early postnatal period by changing the contribution of different types of inhibitory neurons. To test this hypothesis, we first investigated the composition of the population of inspiratory neurons in the preBötC between p1 and p10. In medullary slices prepared from GlyT2-EGFP and GAD65-tdTomato double TG mice, we classified four types of inspiratory neurons: glycinergic, GABAergic, and GGCN by fluorescent protein (FP) expression, and putative excitatory inspiratory neurons by the absence of FP expression^14^. We quantified the shares of these four neuron types among the population of inspiratory neurons, which were identified by applying calcium imaging to visualize Ca^2+^ transients in the inspiratory phase of the breathing cycle. Additionally, we explored the possibility of functional changes in inspiratory neurons during the early postnatal period on the basis of the neuronal activity pattern. For evaluation of the neuronal activity pattern, we used the maximum normalized cross-correlation coefficient (maxCC) between intracellular calcium fluctuations in inspiratory neurons and the local field potential (LFP) of the preBötC. Earlier, we found that the maxCC value of inspiratory neurons was related to the amplitude of intracellular calcium concentration, reflecting the number of action potentials and a given position in the activation sequence during the respiratory rhythmic burst^14^. Combined with the analysis of the maxCC of inspiratory neurons at each postnatal stage, we conclude that the inspiratory neuronal network in the preBötC matures during early postnatal development to become robust.

## Results

### Detection of genetically different types of inspiratory neurons in the preBötC

**Figure 1:**
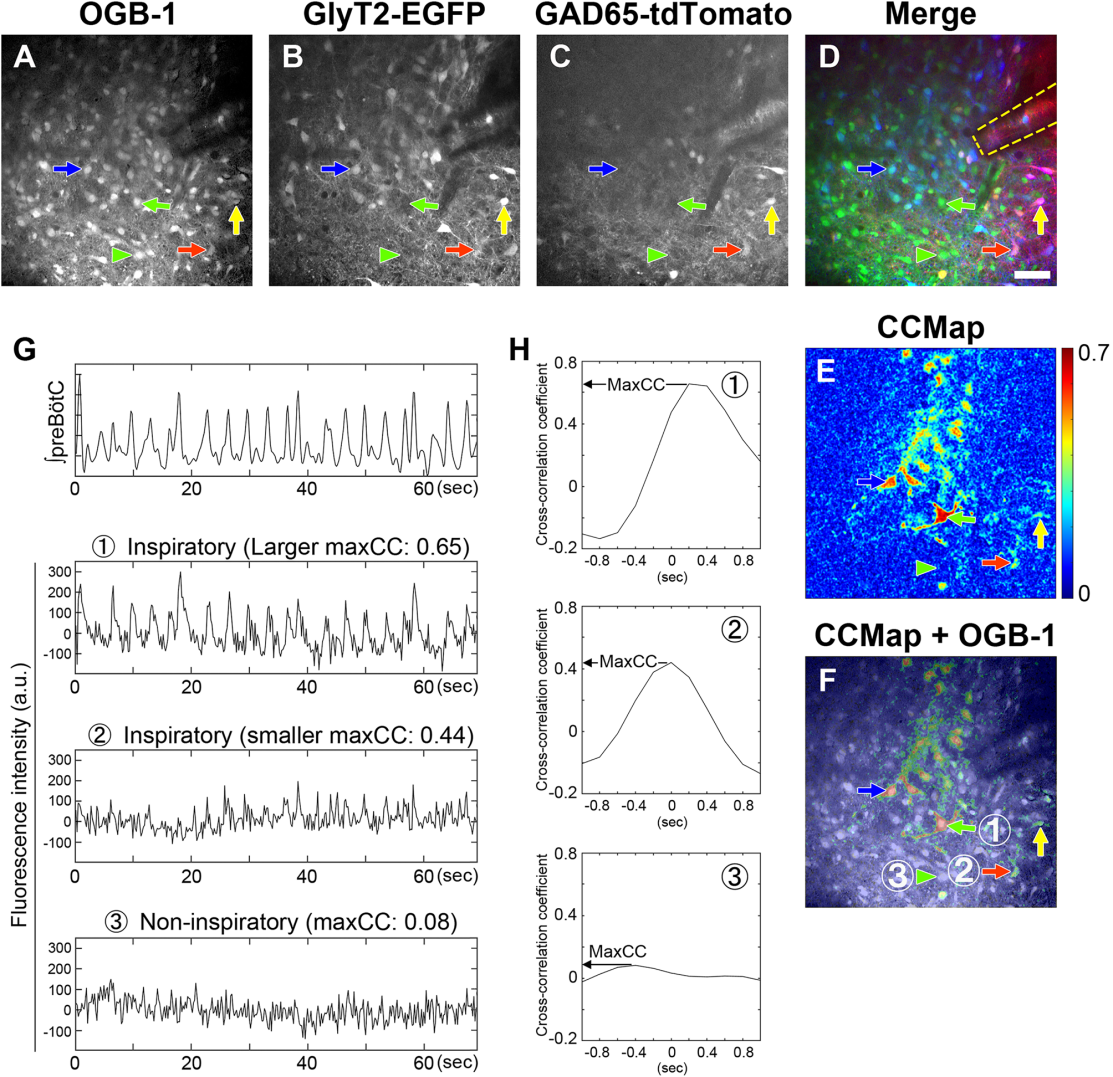
Genetic discrimination of inspiratory neurons in the preBötC using GlyT2-EGFP and GAD65-tdTomato double TG mice at p4. (**A–D)** Fluorescence images of preBötC in slice preparation. (**A**) OGB-1 in all cells, (**B**) EGFP in GlyT2^+^ neurons and (**C**) tdTomato in GAD65^+^ neurons were visualized. (**D**) Overlay image of (**A–C**). Four representative types of cells are indicated by coloured arrows/arrowheads: blue, glycinergic neurons (GlyT2^+^/GAD65^−^); red, GABAergic neurons (GlyT2^−^/GAD65^+^); yellow, GGCN (GlyT2^+^/GAD65^+^, potential dual transmitting neurons); green, other cells, including excitatory neurons (GlyT2^−^/GAD65^−^). (**E**) Cross-correlation (CC) mapping shows the maximum cross-correlation coefficient at individual pixels between the fluctuation pattern of OGB-1 fluorescence and the waveform pattern of integrated LFP. (**F**) CCmap (**E**) overlaid on (**A**). In (**A–F)**, arrows and arrowheads indicate typical inspiratory and noninspiratory neurons, respectively. The outline of the glass suction electrode for recording LFP is denoted by a yellow dashed line in (**D**). The traces of the cells labelled by the circled numbers in (**F**) are shown in (**G,H)**. The scale bar in (**D)** is 50 µm and applies to (**A–F)**. (**G**) Top trace: integrated LFP. From second top to bottom traces: raw traces of simultaneous OGB-1 fluorescence intensity in inspiratory neurons with larger and smaller maxCC or noninspiratory neurons, marked with the circled numbers in (**F)**, respectively. Fluorescence intensity was measured within circles of radius 4 pixels (3.9 µm). (**H**) Each graph indicates the cross-correlation coefficient function calculated between the integrated LFP pattern and the time-shifted OGB-1 fluctuation pattern of each neuron shown by the circled number in (**F**). The maximum value within a time lag between −5 and 5 frames (approximately −1 and 1 s) was the maxCC of each neuron.

To classify inspiratory neurons genetically, we utilized transverse rhythmic slices, including the preBötC, from double TG mice expressing EGFP in GlyT2^+^ neurons and tdTomato in GAD65^+^ neurons. Three types of neurons could be identified using rhythmic slices on the basis of the expression of fluorescent proteins. Glycinergic (GlyT2^+^/GAD65^−^), GABAergic (GlyT2^−^/GAD65^+^), or presumed GABA-glycine cotransmitting (GGCN: GlyT2^+^/GAD65^+^) neurons expressed only EGFP, only tdTomato or both fluorescent proteins, respectively (Fig. 1B–D^14–16^). To detect inspiratory neurons, we focused on the fluctuation pattern of the intracellular calcium concentration, which depends on the number of action potentials^12^. Inspiratory rhythmic bursts arise as a result of action potentials firing from the population of inspiratory neurons in the preBötC. Then, inspiratory activity can be recorded as LFP, reflecting neuronal population activity directly from the preBötC^17^. A cell exhibiting a fluctuation pattern of OGB-1 fluorescence similar to a pattern of simultaneously recorded LFP generally has a large maxCC. Thus, we initially detected prospective inspiratory neurons having greater maxCC than the preset threshold (maxCC = 0.1) by screening using CCmap at each focal plane (Fig. 1E,F). The preset maxCC threshold was low enough to detect all inspiratory neurons and some noninspiratory neurons. Occasionally, cells with large maxCC showed completely different fluctuation patterns of OGB-1 fluorescence from the pattern of LFP for unknown reasons. Therefore, we subsequently identified inspiratory neurons by visual confirmation only if the screen-detected neurons showed peaks of OGB-1 fluorescence intensity during more than 50% of the respiratory cycles (Fig. 1G). Finally, we determined the type of inspiratory neuron by the expression of fluorescent proteins at the positions where inspiratory neurons were detected (Fig. 1A–F). An inspiratory neuron without any expression of EGFP or tdTomato was additionally classified as a putative excitatory inspiratory neuron (Fig. 1A–F; GlyT2^–^/GAD65^−^ indicated by green arrow). To avoid multiple counting of the same inspiratory neuron, we counted inspiratory neurons that were imaged at more than one focal plane as a single neuron.

### Developmental change in the composition of the population of inspiratory neurons

We quantified the shares of the different types of inspiratory neurons at different time points during early postnatal development. The mice were divided into four age groups consisting of postnatal days p1–2, p3–4, p5–6 and p9–10.

**Figure 2:**
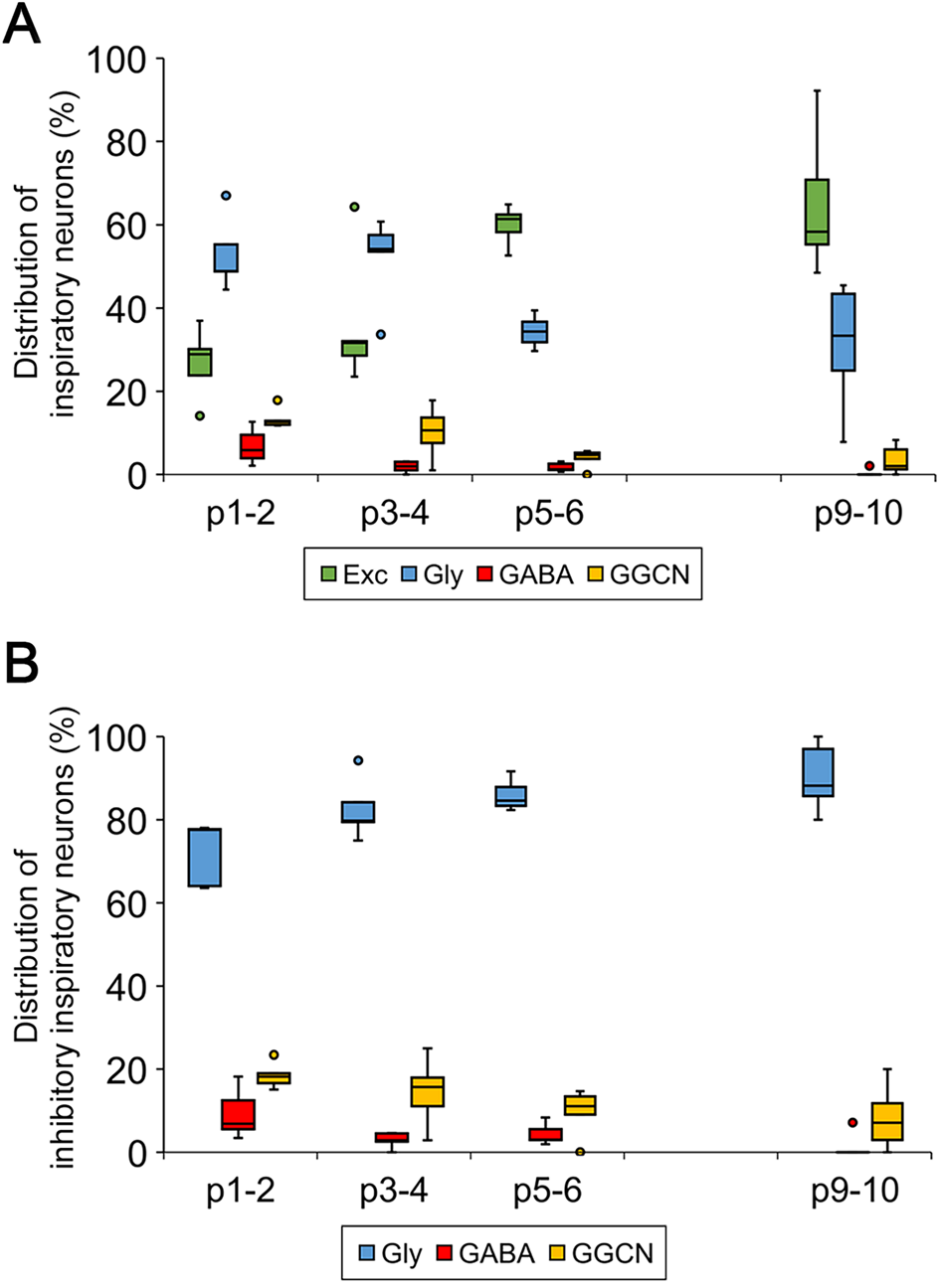
Composition change in the inspiratory neuron population during early postnatal development. Box-and-whisker plots show composition rates of respective types of inspiratory neurons over total inspiratory neurons (**A**) or total inhibitory inspiratory neurons (**B**) at each age. The line in the box indicates the median value in each group. The upper and lower edges of the box show the 75th and 25th percentiles in each group, respectively. Both ends of the whisker show the maximum and the minimum values, excluding any outliers in each group. The coloured circle indicates the outlier. All results were calculated using the shares of respective types of inspiratory neurons from five rhythmic slices (n = 5 in each age group).

In the first two age groups (p1–2 and p3–4), the major type of inspiratory neurons was glycinergic neurons, accounting for 53% (44–67%; n = 5) and 52% (34–61%; n = 5) of the total number of inspiratory neurons, respectively (Fig. 2A). Putative excitatory inspiratory neurons formed the second largest population for the same periods (Fig. 2A: 27%; 14–37% and 36%; 24–64% at p1–2 and p3–4; n = 5, respectively).

In the third and last age groups (p5–6 and p9–10), the relative ranking of the percentages between putative excitatory and glycinergic inspiratory neuron types inverted (Fig. 2A). The averaged percentages of putative excitatory inspiratory neurons greatly increased at p5–6 (60%; 53–65%; n = 5) and p9–10 (65%; 49–92%; n = 5) compared to the previous two age groups. In contrast, the percentages of glycinergic inspiratory neurons decreased at p5–6 (34%; 30–40%; n = 5) and p9–10 (31%; 8–46%; n = 5).

The share of GGCN among total inspiratory neuron populations changed in a similar pattern in comparison with glycinergic inspiratory neurons, while the mean values were lower. The percentages of inspiratory GGCN decreased from p1–2 (14%; 12–18%; n = 5) to the latter two groups, p5–6 (4%; 0–6%; n = 5) and p9–10 (3%; 0–8%; n = 5) (Fig. 2A). GABAergic inspiratory neurons formed the smallest population among the four types of inspiratory neurons from p1 to p10. The percentage of GABAergic inspiratory neurons among total inspiratory neurons at p1–2 (7%; 2–13%; n = 5) further decreased even at p3–4 (2%; 0–3%; n = 5) (Fig. 2A). Then, we conducted Pearson’s chi-square test using the total number of inspiratory neurons in each group divided by age and neuron type from five rhythmic slices (Table 1). The compositions of the population of inspiratory neurons were significantly different among age groups (*P* < 0.0001). These results indicate that putative excitatory inspiratory neurons driving rhythmic bursts would become the principal population in the inspiratory neuronal network in the preBötC at p5-6, although glycinergic inspiratory neurons are the major population at birth. Consequently, the inspiratory neuronal network in the preBötC might develop and mature at the level of its neuronal populations during the early postnatal period.

**Table 1:** Total number of inspiratory neurons.

Neuron type	Age				Total
	P1–2	P3–4	P5–6	P9–10	
Excitatory	107	175	236	179	697
Glycinergic	213	241	127	81	662
GABAnergic	26	11	5	1	43
GGCN	54	39	17	8	118
Total (inhibitory)	400 (293)	466 (291)	385 (149)	269 (90)	1520 (823)

Data are shown as the total number of each type of inspiratory neurons from five rhythmic slices at each age group. The numbers in parentheses at the lowest row indicate the total number of inhibitory inspiratory neurons at each age. Both in the total inspiratory neuron group and total inhibitory inspiratory neuron group, the composition of inspiratory neurons were significantly different among respective age groups (*P* < 0.05; Pearson’s Chi-square test).

Next, we quantified the shares of the three types of inhibitory neurons among the total number of inhibitory inspiratory neurons at each stage. As shown in Fig. 2B, the proportion of glycinergic inspiratory neurons showed a steady increase during early postnatal development (72%; 64–78%, 83%; 75–94%, 86%; 83–92% and 90%; 80–100% at p1–2, p3–4, p5–6 and p9–10; n = 5). The percentages of inspiratory GABAergic neurons and inspiratory GGCN, both of which expressed GAD65, decreased with age (Fig. 2B). The composition of the population of inhibitory inspiratory neurons were significantly different among age groups (*P* = 0.0009; Pearson’s Chi-square test). Since GABAergic neurons and GGCN gradually lost their synchronicity with rhythmic bursts and became minor neuron types early after birth, we considered that these neurons might contribute to rhythm modulation by asynchronous activities such as tonic activities rather than to rhythm generation by synchronous activities, at least in mice older than four days. Taken together, putative excitatory and glycinergic inspiratory neurons might serve central roles in inspiratory pattern and/or rhythm generation in the preBötC through their synchronous activities, at least after maturation.

### Possibility of developmental change in the activity-related functions of inspiratory neurons

**Figure 3:**
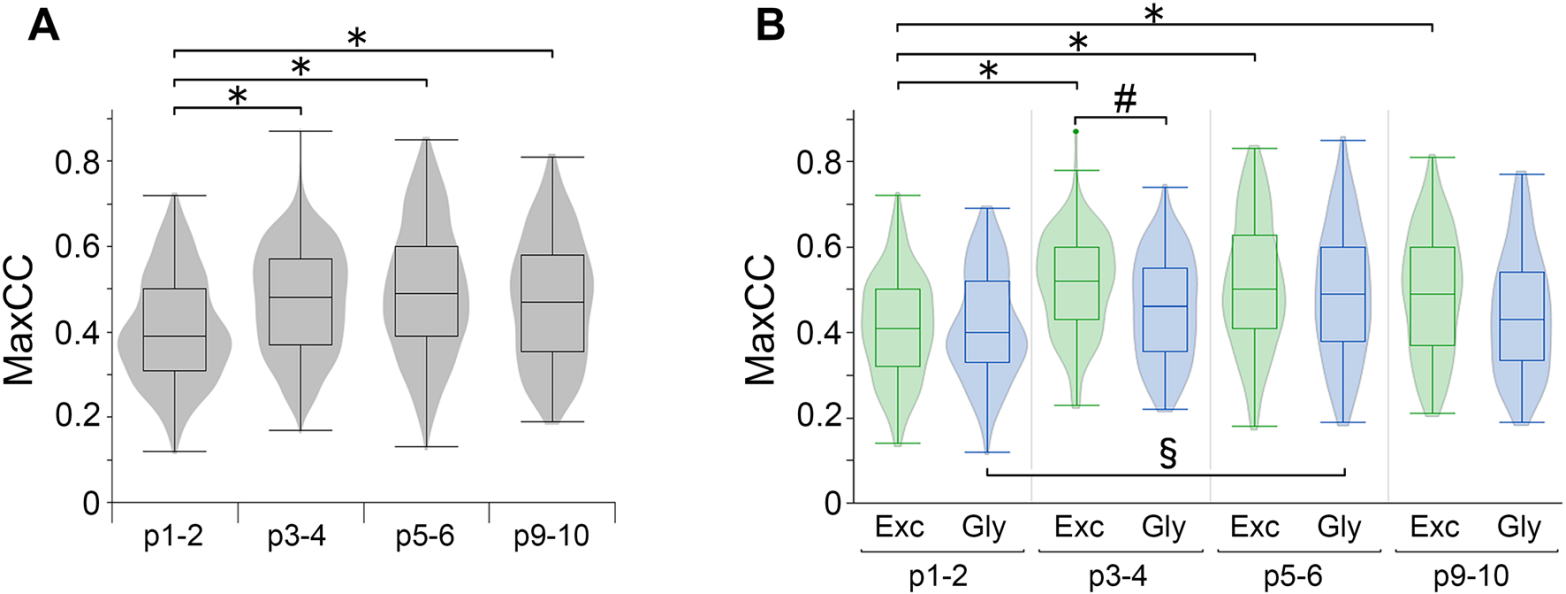
Differences in the relationship of activity patterns of inspiratory neurons to rhythmic bursting patterns of the preBötC during early postnatal development. MaxCC is used as an index to reflect the number and peak timing of action potentials firing on inspiratory neurons. Previously, we showed that inspiratory neurons with larger maxCC exhibited clear waveforms of intracellular calcium rises later in the activation sequence during rhythmic burst. (**A**) Violin plots with box-and-whisker plots show the probability density distribution and the median maxCC of all inspiratory neurons at respective ages. The plots were calculated from 400, 466, 385, and 269 cells detected from *n* = *5* mice at each time point from p1–2 to p9–10. Significant differences were evaluated among age groups (*p < 0.05 vs. p1–2, Kruskal‒Wallis one-way ANOVA). (**B**) Violin plots with box-and-whisker plots show the probability density distribution and the median maxCC of putative excitatory and glycinergic inspiratory neurons at respective ages. The plots were calculated from 107, 175, 236, and 179 putative excitatory inspiratory neurons and 213, 241, 127, and 81 glycinergic inspiratory neurons, which were detected from *n* = *5* mice at each time point from p1–2 to p9–10. Significant differences were evaluated among all combinations of populations divided by ages and neuron types. Only the differences between neuron types per age group and the differences among age groups per neuron type are shown (*p* < 0.05 vs. p1–2 of putative excitatory inspiratory neuron type (*), p1–2 of glycinergic inspiratory neuron type (§) and inspiratory putative excitatory neuron type per age group (#), two-way ANOVA). In both (**A,B**), the line in the box shows the median maxCC; the upper and lower edges of the box show the 75th and 25th percentiles of the maxCC; and both ends of the whisker show the maximum and the minimum maxCC in each group.

Previously, we further classified inspiratory neurons into two groups on the basis of the maxCC, which is an index value indicating how similar the fluctuation pattern of OGB-1 fluorescence in neurons is to the pattern of LFP^14^. The magnitude of maxCC is influenced by the patterns of neuronal activities. We proposed that one group of inspiratory neurons with a smaller maxCC would play a leading role in the generation of rhythmic bursts, while another group of inspiratory neurons with a larger maxCC could be subsequently activated to generate high-amplitude inspiratory bursts. Next, to assess the possibility of functional changes in inspiratory neurons during early postnatal development, we investigated how the maxCC of inspiratory neurons changed with growth. First, we studied the differences in the maxCC of all inspiratory neurons among age groups. The median maxCC in the p1–2 group (0.39; n = 400) was smaller than those in the other age groups (0.47–0.49; n = 466, 385 and 269 at p3–4, p5–6, and p9–10, respectively) (Table 2). To visualize the distribution of maxCC of inspiratory neurons, we generated violin plots of maxCC in addition to box-and-whisker plots (Fig. 3). The violin plot displays the probability density function of the data, which is estimated non-parametrically using a kernel density estimation, symmetrically across the centre line. The violin plot is useful to compare distributions of data across different groups because the plots can show the peak position and relative amplitude of the distribution precisely^18^. The probability density distributions of the maxCC of all inspiratory neurons obviously differed between p1–2 and the other age groups. For example, one large peak was observed at a lower maxCC range (approximately 0.39) in the violin plot at p1–2 (Fig. 3A). Thus, the maxCC of all inspiratory neurons at p1–2 was significantly different from those of the other three age groups (*P* < 0.001, p1–2 vs. respective groups), and there were no significant differences among any other combinations of age groups. These results suggest that the leading type forming a majority of the inspiratory neuron population at birth might change their activity patterns during early postnatal development. In any case, inspiratory neurons well-synchronized with rhythmic bursts would have increased their shares in the inspiratory neuronal network in the preBötC from p3–4.

**Table 2:** Median maxCC of inspiratory neurons.

Neuron type	p1–2	p3–4	p5–6	p9–10
All	0.39	0.48	0.49	0.47
Excitatory	0.41	0.52	0.50	0.49
Glycinergic	0.40	0.46	0.49	0.43

Data are shown as median maxCC of inspiratory neurons belonging to each neuron type. Group of “all” includes inspiratory excitatory, glycinergic, GABAergic and GGC neurons.

Next, we examined the differences in the maxCC of each individual inspiratory neuron type. Considering the very small shares of inspiratory GABAergic and GGC neurons, we assumed that they might minimally influence the rhythmic activity generated in the inspiratory neuronal network in the preBötC. Therefore, we focused only on putative excitatory and glycinergic inspiratory neurons. In both putative excitatory and glycinergic inspiratory neurons, the median maxCC in the p1–2 group (excitatory: 0.41; n = 107, glycinergic: 0.40; n = 213) was smaller than those in the other age groups (excitatory: 0.49–0.52, glycinergic 0.43–0.49) (Table 2). Furthermore, the shapes of violin plots of maxCC for p1–2 looked completely different from those for the other three ages for both inspiratory neuron types (Fig. 3B). In putative excitatory inspiratory neurons, significant differences in maxCC were detected only between p1–2 and each of the other three age groups (*P* < 0.001, p1–2 vs. respective groups). In glycinergic inspiratory neurons, maxCC was significantly different only between the p1–2 and p5–6 groups (*P* < 0.001). In the same age groups, a significant difference in maxCC between excitatory and glycinergic inspiratory neuron types was detected only at p3–4 (*P* = 0.0004). Over all age groups, the distribution of maxCC was not obviously different between these two types of inspiratory neurons, although the median values of maxCC of glycinergic inspiratory neurons were slightly smaller than those of putative excitatory inspiratory neurons (Table 2). Thus, the maxCC values of both inspiratory neuron types started to increase from p3, although this shift appeared to be delayed in the glycinergic inspiratory neuron type compared to the putative excitatory inspiratory neuron type. Considering the drastic increase in the share of excitatory inspiratory neurons among total inspiratory neurons at p5–6 (Fig. 2A), the inspiratory neuronal network in the preBötC might have changed to contain a larger population of putative excitatory inspiratory neurons well-synchronized with rhythmic bursts at p3–6. These results suggest that the inspiratory neuronal network might acquire both a sufficient share of excitatory inspiratory neurons, which provide a driving force for the generation of high-amplitude inspiratory bursts, and a well-balanced share of glycinergic inspiratory neurons, of which coactivation is essential for gain control for the excitatory inspiratory neurons within the first postnatal week. Consequently, the inspiratory neuronal network might mature to generate clearer and stronger inspiratory motor output during early postnatal development.

## Discussion

In this study, we evaluated whether and how the inspiratory neuronal network in the preBötC changes during early postnatal development. To the best of our knowledge, our work provides the first examples of early postnatal changes in both the composition of the population of inspiratory neurons and the trend of synchronous neuronal activity with spontaneous inspiratory rhythmic bursts. Surprisingly, the developmental change in the composition of the inhibitory inspiratory neuron population was different from that of the total inhibitory neuron population, including noninspiratory neurons in the preBötC^8^. In the present study, glycinergic inspiratory neurons were the major cell type among inhibitory inspiratory neurons at p1–2, and their fraction continued to increase until p9-10. Conversely, the fraction of GABA-releasing inspiratory neurons, both GABAergic and GGC inspiratory neurons, among inhibitory inspiratory neurons decreased after birth (Fig. 2B). However, within the total population of inhibitory neurons in the preBötC (i.e., not only inspiratory neurons), GGCN represented the major component, and glycinergic neurons formed the smallest population before p5 (GGCN: 53% and 44% at p0 and p4, glycinergic neurons: 20% and 27% at p0 and p4)^8^. GABAergic neurons as well as glycinergic neurons increased from birth to approximately p10 (GABAergic neurons: 28%, 30% and 33% at p0, p4 and p8, glycinergic neurons: 45% at p8), although GGCN decreased from the embryonic period^8^. The shares of the three inhibitory neuron types among the inhibitory inspiratory neuron population appeared to differ from the shares among the total inhibitory neuron population. However, the reason for the difference in the share percentages between the two populations could be explained by calculation using the share of inspiratory neurons among each inhibitory neuron type, especially before p5. In previous reports, GABA-releasing neurons expressing GAD65 and glycine-releasing neurons expressing GlyT2 accounted for 70–80% and approximately 70% of the total inhibitory neuron population at p0–4, respectively^8^. In p0–4 mice, approximately 20% of total GABA-releasing neurons were inspiratory neurons, although the marker for GABA-releasing neurons was GAD67 instead of GAD65^9^. Inspiratory-type GABA-releasing neurons composed approximately 15% (70–80% × 20%) of the total inhibitory neuron population at p0–4. Similarly, 42% of all glycine-releasing neurons were inspiratory neurons in p0–7 mice^10^. Given that the share of inspiratory neurons in all glycine-releasing neurons was 42% at p0–4 as well as at p0–7, glycine-releasing inspiratory neurons would comprise approximately 30% (72% × 42%) of the total inhibitory neuron population at p0-4. Thus, the ratio of the number of GABA-releasing inspiratory neurons to glycine-releasing inspiratory neurons would be 1:2 (15:30) at p0–4. Because GGCN were counted in both groups of GABA-releasing and glycine-releasing neurons, glycinergic inspiratory neurons would theoretically compose 50–67% of the inhibitory inspiratory neuron population at p0–4. Furthermore, glycinergic inspiratory neurons might compose a larger percentage of inhibitory inspiratory neuron populations at p0–4 because the share percentage of inspiratory neurons in the population of glycine-releasing neurons decreased to 20% at p4–7^12^ compared with 42% at p0–7. Therefore, glycinergic inspiratory neurons could form the majority of the inhibitory inspiratory neuron population, as we indicated in this study. Furthermore, the differentiation of GGCN to glycinergic neurons started around p4, and consequently, the share of GGCN decreased, but that of glycinergic neurons increased among the population of total inhibitory neurons in the preBötC^8^. This shift towards glycinergic neurons would support our present results of increasing share of glycinergic inspiratory neurons among the inhibitory inspiratory neuron population. Further studies are expected to reveal the composition rates of inspiratory neurons per neuron type during early postnatal development.

We assumed that the gap in compositions between the inhibitory inspiratory neuron population and the total inhibitory neuron population might result from the differences in the neuronal functions for inspiratory rhythm formation. In the slice preparation, glycinergic but not GABAergic inhibitory inputs inhibited the tonic activities of expiratory neurons during rhythmic bursts periodically^12,19^. Thus, some glycinergic neurons are supposed to work as inspiratory neurons to inhibit expiratory neurons for a part of inspiratory rhythm generation. Moreover, both inspiration-related glycinergic inhibitory inputs and excitatory inputs were recorded on other preBötC glycinergic neurons simultaneously, which may contribute to inspiratory pattern generation^12^. Therefore, a number of glycinergic inspiratory neurons could be incorporated into the inspiratory neuronal network of the preBötC. In contrast, the physiological role of GABA-releasing inspiratory neurons remains unclear. Many pharmacological and optogenetic experiments have demonstrated that GABA is an important inhibitory neurotransmitter that modulates the inspiratory rhythm in the preBötC^20–23^. Nevertheless, since the activities of both inspiratory and noninspiratory neurons were uniformly controlled using pharmacological and optogenetic methods, the functions of GABA-releasing inspiratory neurons alone have yet to be determined. Because of the small total number of GABA-releasing inspiratory neurons in our studies, we suppose that GABA-releasing neurons might modulate inspiratory rhythm by their noninspiratory activities, such as tonic activities, rather than rhythmic activities and that phasic inputs from inspiratory-type GABA-releasing neurons might manifest different roles until p4.

Excess GABA inhibited respiratory rhythm generation in brainstem-spinal cord preparations from E18.5 foetuses and slice preparations from p0–3 mice^13,24^. GABA would mainly work as an inhibitory neurotransmitter, at least at GABA_B_ receptors in the preBötC of mouse brain slices, for respiratory pattern and/or rhythm generation even in the perinatal period^13^. Thus, intracellular chloride ion concentration ([Cl^−^]_i_) would be low in respiratory-related neurons in the perinatal period^24^. However, the reversal potential of the GABA_A_-receptor-activated membrane response (E_GABA-A_) in slice preparations changed from depolarizing to hyperpolarizing at approximately p4, after which the share of GABA-releasing inspiratory neurons drastically decreased^11^. Additionally, GAD67-knockout mice died within 6 h after birth because of respiratory failure, and an abnormal respiratory pattern was observed both in vivo and in brainstem-spinal cord preparations^25^. Therefore, one can speculate that phasic-released GABA might serve as an excitatory neurotransmitter at a part of GABA_A_ receptors for the development of the inspiratory neuronal network, at least within the prenatal period or up to p4. This contradictory effect of GABA might be attributed to the differences of the local [Cl^−^]_i_. Actually, the alpha2 subunit of Na^+^, K^+^-ATPase colocalizes with KCC2 on the synaptic membrane in the neuron and generates a local intracellular K^+^ gradient, which could drive the extrusion of Cl^−^ by the colocalized KCC2 resulting in the local low [Cl^−^]_i_^24^. In this case, GABAergic and glycinergic inputs could cause hyperpolarizing potentials at the synapse where KCC2 colocalized with the alpha2 subunit of Na^+^, K^+^-ATPase. Conversely, GABA might also cause depolarization if other GABAergic presynaptic neuronal processes would terminate on regions of dendrites with higher [Cl^-^]_i_, e.g., because the local function of NKCC1 is dominant over that of KCC2 in the postsynaptic neuron. Further investigations are expected to reveal the relationship between the local [Cl^−^]_i_ in the neuron and other local synaptic features such as, e.g., the expression of neurotransmitter receptors and ion transporters, for understanding of the perinatal and early postnatal developmental change of the inspiratory neuronal network in the preBötC.

We further found that putative excitatory inspiratory neurons became the largest fraction of neurons among the total inspiratory neuron population from p5–6. Since excitatory inspiratory neurons are necessary to generate the inspiratory rhythm^21^, this change might be related to the increase in respiratory frequency and the stabilization of breathing parameters during the early postnatal period^6,7^. Moreover, the median and distribution of the maxCC values of putative excitatory inspiratory neurons with respect to the LFP of the preBötC shifted towards significantly larger values at p3–4 from p1–2. We previously indicated that inspiratory neurons with large maxCC values displayed intracellular calcium fluctuation waveforms with large amplitudes and longer durations during inspiratory rhythmic bursts; in contrast, inspiratory neurons with small maxCC values exhibited smaller and shorter calcium signal waveforms^14^ (Fig. 1G). Furthermore, inspiratory neurons exhibiting peaks of their calcium transients earlier in the activation sequence during rhythmic bursts had small maxCC values, and inspiratory neurons with larger maxCC values exhibited peaks later in the sequence. Since fluctuations in the intracellular calcium concentration depend on the number of action potentials^12^, we previously suggested that excitatory inspiratory neurons with small maxCC, which are stochastically fired with a small number of action potentials, might lead to inspiratory rhythm generation by activating excitatory inspiratory neurons with large maxCC, which could percolate to generate high-amplitude inspiratory bursts^14^. Therefore, the postnatal increase in the share of putative excitatory inspiratory neurons with large maxCC might provide a sufficient driving force to the inspiratory neuronal network for the generation of a stable inspiratory rhythm. The maxCC of glycinergic inspiratory neurons also shifted towards larger values, although with some delay compared to excitatory inspiratory neurons. In accordance with the change in the activity pattern, glycinergic inspiratory neurons could acquire the ability to provide well-balanced gain control for the excitatory inspiratory neurons. These early postnatal property changes of inspiratory neurons might contribute to the formation of the robust inspiratory neuronal network in the preBötC. Further investigation of the developmental changes in neuronal properties and functions in the process of inspiratory rhythm generation shall be interesting for understanding the development of the inspiratory neuronal network.

### Technical considerations

In this study, we applied calcium imaging in slice preparations from mouse brains to detect as many inspiratory neurons as possible. Formally, we cannot exclude the possible existence of inspiratory neurons that fire action potentials during inspiration bursts without increasing somatic intracellular calcium.

We classified the types of neurons according to the expression of fluorescent proteins in transgenic mice. Thus, our results would contain some “time smearing” of neuron type development because of the time delay of both the expression and degradation of the fluorescent proteins, as described in detail previously^8^. This might result in the delay of the increase of the fraction of putative excitatory inspiratory neurons at p4–5 to a time point after the larger shift of maxCC values of the neurons at p3–4. Nevertheless, since immunohistochemistry, another common method for cell classification, has the major limitation of the substantial challenge of relating cell identity to cellular activity during inspiration assessed by calcium imaging in live tissues, we consider our method to provide a useful approach.

In our study, GlyT2^−^/GAD65^−^ neurons were defined as putative excitatory neurons. Principally, the population of GlyT2^−^/GAD65^−^ neurons might, however, include GAD65^−^/GAD67^+^ neurons or glial cells. Both GAD65 and GAD67 coexisted in GABA-releasing neurons, although the localization inside the neurons was shown to differ between the two isoforms^26^. Somatic intracellular calcium fluctuation in glial cells is generally much slower than that in neurons. However, to assure the developmental composition change of excitatory inspiratory neurons, further investigation using markers for an excitatory neuron, e.g., vesicular glutamate transporter (Vglut) or developing brain homeobox protein 1 (Dbx1), must be considered.

Follow-up studies to conduct more detailed investigation of the changes in the properties of inspiratory neurons during the early postnatal period are recommended. As an attractive challenge, how the composition of the population of inspiratory neurons in the preBötC changes during the prenatal period should also be investigated. It is expected that these studies can provide further insight into the development of the inspiratory neuronal network in the preBötC and the mechanism of some central breathing problems, such as apnoea of prematurity.

## Conclusions

We conclude that the inspiratory neuronal network in the preBötC develops at the level of both neuronal populations and neuronal activities during the early postnatal period. We found that the composition of the population of inspiratory neurons drastically changed from p0 to p10, and the activity patterns of excitatory as well as glycinergic inspiratory neurons became well-synchronized with the inspiratory rhythmic bursting pattern in the preBötC within the first postnatal week. The early postnatal development of inspiratory neurons might support the robustness of the inspiratory neuronal network in the preBötC for lifelong breathing activity.

## Methods

### Ethics approval

This study was carried out in accordance with the ARRIVE guidelines, with the guidelines for the welfare of experimental animals issued by the European Communities Council Directive 2010/63/EU and with the German Protection of Animals Act (TierSchG). All experiments and animal care were conducted in agreement with § 4 Abs. 3 TierSchG and were approved and registered (T12/11) by the animal welfare office and commission of the University Medical Center Göttingen.

### Animals

Mice were bred in the animal facility of the University Medical Center Göttingen. We crossbred Tg(Gad2-tdTomato)DJhi mice expressing the red fluorescent protein tdTomato under the GAD-65 promoter in GABAergic neurons^15^ with Tg(Scl6a5-EGFP)1Uze mice expressing enhanced green fluorescent protein (EGFP) under the GlyT2 promoter in glycinergic neurons^16^. Offspring were housed with their mothers under a 12:12 h light/dark cycle. Twenty healthy neonatal mice were randomly allocated to four groups according to their age on days p1–2, p3–4, p5–6 and p9–10 (*n* = 5 in each group).

### Rhythmic slice preparation

Rhythmic slices were prepared from mice between postnatal days 1–10 as described previously^14^. Briefly, mice were deeply anaesthetized with isoflurane and decapitated. The brainstem was rapidly isolated and mounted on an agar block, which was prepared by dissolving 4 wt.% of agar (Sigma-Aldrich Co., MO, USA) in distilled water, under the condition of ice-cold, oxygenated (95% O_2_, 5% CO_2_) artificial cerebrospinal fluid (aCSF) composed of (in mM): 118 NaCl, 3 KCl, 1.5 CaCl_2_, 1 MgCl_2_, 1 NaH_2_PO_4_, 25 NaHCO_3_ and 30 D-glucose (pH 7.4). The agar was set on a vibroslicer (Leica VT 1200S, Leica Biosystems, Nussloch, Germany). Transverse slices were cut in a caudal-rostral direction step-by-step until the area postrema was visible under the condition of ice-cold, oxygenated high osmolality Ringer’s solution without calcium composed of (in mM): 124 NaCl, 3 KCl, 2 MgCl_2_, 1.3 NaH_2_PO_4_, 26 NaHCO_3_, 10 d-glucose, 200 sucrose and 1 kynurenic acid (pH 7.4)^27^. Then, a transverse slice (550–600 µm thickness) was prepared from the brainstem. The slice contained the preBötC where the rhythmic population of neurons was detected on the surface of the rostral section. To induce rhythmic activity, each slice was superfused in aCSF at 28 °C with 8 mM KCl at a flow rate of 4 mL/min^2^.

Local field potential (LFP) was recorded from the preBötC using glass suction electrodes filled with the aCSF^14^. The internal diameter of the glass suction electrodes was about 25 µm. The electrical signals were amplified 5000–200,000 times, bandpass filtered (0.25–3.5 kHz) and digitized at 10 kHz on a Digidata interface using pClamp10 software (Molecular Devices Inc., CA, USA). Recordings were rectified and integrated online (time constant = 100–200 ms).

### Calcium imaging and two-photon microscopy recording

Cells in the preBötC were labelled by injection of the calcium indicator dye Oregon Green 488 BAPTA-1 AM (OGB-1, Thermo Fisher Scientific Inc., MA, USA) into the slice^14,28^. The OGB-1 stock solution was prepared by dissolving 50 µg of OGB-1 in 40 µL of DMSO containing 20% Pluronic F-127 (Thermo Fisher Scientific Inc., MA, USA) and stored at −20 °C in 4 µL aliquots before use. One aliquot of the OGB-1 stock solution was diluted with 16 µL of a pipette solution containing (in mM) 150 NaCl, 2.5 KCl, and 10 HEPES (pH 7.4) to prepare an injection solution at a final OGB-1 concentration of 200 µM. OGB-1 was pressure-injected for 10 min (70 kPa) using a glass pipette into the same side of the preBötC where LFP was recorded. Afterwards, the slice was superfused with aCSF for more than 40 min to wash out excess dye before starting imaging.

We acquired all fluorescence images using a 2-photon laser-scanning microscope (TriMScope, LaVision, BioTec, Bielefeld, Germany) equipped with a 20× (1.0 NA) water immersion objective lens (Zeiss, Oberkochen, Germany) and a nondescanned detector equipped with GaAsP photomultipliers (Hamamatsu Photonics K.K., Hamamatsu, Japan). Two-photon excitation of tdTomato, OGB-1 or EGFP was achieved with a Ti:Sapphire Laser (MaiTai BB, SpectraPhysics, CA, USA) at 720 nm, 800 nm or 900 nm, respectively. Emitted fluorescence was detected through three kinds of bandpass emission filters: 641/75 nm for tdTomato, 531/40 nm for OGB-1 or 475/50 nm for EGFP (AHF Analysentechnik AG, Tübingen, Germany)^12,14,29^. Time series of the calcium signal and reference still images for tdTomato, OGB-1 and EGFP were recorded at several depths between 0 and 120 µm from the surface of the slice. The sizes of the recorded fields ranged from 221 × 250 µm (226 × 256 pixels) to 350 × 350 µm (359 × 359 pixels). Calcium imaging data were captured at a frame rate of approximately 5 or 10 Hz. The sampling frequency varied slightly depending on the data due to the characteristics of the measurement equipment. The data acquired at approximately 10 Hz were compressed to an approximately 5 Hz frame rate for statistical analysis (see “Data processing” section). During the acquisition of calcium imaging data, the triggered pulse for each frame was output to the LFP recording system and recorded. All settings were controlled using “Imspector Pro” software (LaVision, BioTec, Bielefeld, Germany).

### Data processing

All fluorescence images were saved in TIFF format. Reference still images for three fluorophores were preprocessed by using ImageJ (http://rsb.info.nih.gov/ij/). Since fluorescence from the three fluorophores exhibits some spectral overlap, we decomposed the overlapping signals and isolated the signals from the respective fluorophores by nonnegative tensor factorization using a spectral unmixing plug-in for ImageJ^30^. We classified cell types genetically according to the isolated fluorescence signals. All other data processing was performed using MATLAB (MathWorks, MA, USA). At the beginning of preprocessing of calcium imaging data, we discarded the first 100 frames of the data because the fluorescence intensity was unstable shortly after the recording started due to several reasons, including fluorescence photobleaching. Calcium imaging data were registered on the first time frame image using the rigid-body realignment procedure for the correction of sample position misalignment caused by mechanical vibration. Third, the time series of OGB-1 fluorescence at each image pixel was bandpass filtered (0.1–1.0 Hz, 3rd order zero-phase Butterworth filter) and then spatially filtered by taking the unweighted average of a 3 × 3 region around each pixel (2.93 × 2.93 µm). LFP data were bandpass filtered (0.1–1.0 Hz: the same cut-off frequencies as the bandpass filter for calcium imaging). The filtered LFP data were resampled to the same sampling rates as the corresponding calcium imaging using an anti-aliasing method with a low-pass Chebyshev Type I infinite impulse response (IIR) filter of order 8. We defined a rhythmic burst as an event observed only when a peak LFP value was larger than 0.8× the standard deviation of the preprocessed LFP. The maximum normalized cross-correlation coefficient (maxCC) was calculated between a bursting pattern of the preprocessed LFP and a fluctuation pattern of the preprocessed OGB-1 fluorescence for each pixel. Maxima of the cross-correlation coefficients were searched within a time lag between −5 and 5 frames (Fig. 1H) and were plotted on CCmap (Fig. 1E). The cross-correlation coefficient function was defined as:

$${R}_{xy}\left(\tau \right)=\frac{Cov\left(x\left(t\right),y\left(t+\tau \right)\right)}{{\sigma }_{x}{\sigma }_{y}}$$

where $x$ is the LFP and $y$ is signal at each neuron, ${\sigma }_{x}$ and ${\sigma }_{y}$ are standard deviation of $x$ and $y$ respectively. We performed screening for inspiratory neurons using maxCC at the centre of cells on the CCmaps. The threshold for the screening was set at [maxCC = 0.1]. Furthermore, we visually double-checked whether peaks of raw fluorescence fluctuations within circles of radius 4 (3.9 µm) around the centres of cells had been coincident with rhythmic burst signals in the preprocessed LFP. The MaxCC of inspiratory neurons was recalculated for statistical analysis using calcium imaging data and the corresponding LFP data that were subsampled to 5 Hz before spatial filtering if the imaging data were recorded at 10 Hz. The data length used for the recalculation of maxCC was approximately 107 s (approximately 545 frames) to include rhythmic bursts from 11 to 33 cycles. When the respiratory cycles in the data were rapid, we used shorter datasets (at least approximately 75 s: 384 frames) containing rhythmic bursts of between 16 and 30 cycles. Conversely, for data exhibiting slow respiratory cycle rates, longer datasets (approximately 157 s: 800 frames) were used for recalculation.

### Statistical analysis

All significance tests were carried out using SAS OnDemand for Academics (SAS Institute Inc., NC, USA). Boxplots in Fig. 2 were created using Excel (Microsoft, WA, USA). Violin plots in Fig. 3 were generated using JMP Pro 16 (SAS Institute Inc., NC, USA). Significant differences in the composition of the population of the inspiratory neurons among respective age groups were examined by Pearson’s chi-square test. To test significant differences in the maxCC of all inspiratory neurons among age groups, we conducted Kruskal‒Wallis one-way ANOVA followed by Steel–Dwass-Critchlow-Fligner post hoc test. To evaluate significant differences in maxCC among populations divided by age and neuron type, two-way ANOVA followed by Bonferroni’s post hoc test was conducted. Differences were considered statistically significant at *P* < 0.05.

## Data Availability

The datasets used and analysed during the current study are available from the corresponding author upon reasonable request.

## References

[1] Feldman JL, Del Negro CA (2006). Looking for inspiration: New perspectives on respiratory rhythm. Nat. Rev. Neurosci..

[2] Smith JC, Ellenberger HH, Ballanyi K, Richter DW, Feldman JL (1991). Pre-Botzinger complex: A brainstem region that may generate respiratory rhythm in mammals. Science.

[3] Greer JJ, Funk GD, Ballanyi K (2006). Preparing for the first breath: Prenatal maturation of respiratory neural control. J. Physiol..

[4] Pagliardini S, Ren J, Greer JJ (2003). Ontogeny of the pre-Bötzinger complex in perinatal rats. J. Neurosci..

[5] Thoby-Brisson M, Trinh JB, Champagnat J, Fortin G (2005). Emergence of the pre-Botzinger respiratory rhythm generator in the mouse embryo. J. Neurosci..

[6] Hulsmann S (2018). The postnatal development of ultrasonic vocalization-associated breathing is altered in glycine transporter 2-deficient mice. J. Physiol..

[7] Viemari JC, Burnet H, Bévengut M, Hilaire G (2003). Perinatal maturation of the mouse respiratory rhythm-generator in vivo and in vitro studies. Eur. J. Neurosci..

[8] Hirrlinger J (2019). GABA-glycine cotransmitting neurons in the ventrolateral medulla: Development and functional relevance for breathing. Front. Cell. Neurosci..

[9] Kuwana S (2006). Electrophysiological and morphological characteristics of GABAergic respiratory neurons in the mouse pre-Botzinger complex. Eur. J. Neurosci..

[10] Morgado-Valle C, Baca SM, Feldman JL (2010). Glycinergic pacemaker neurons in preBotzinger complex of neonatal mouse. J. Neurosci..

[11] Ritter B, Zhang W (2000). Early postnatal maturation of GABAa-mediated inhibition in the brainstem respiratory rhythm-generating network of the mouse. Eur. J. Neurosci..

[12] Winter SM (2009). Glycinergic interneurons are functionally integrated into the inspiratory network of mouse medullary slices. Pflugers Arch..

[13] Zhang W, Barnbrock A, Gajic S, Pfeiffer A, Ritter B (2002). Differential ontogeny of GABA(B)-receptor-mediated pre- and postsynaptic modulation of GABA and glycine transmission in respiratory rhythm-generating network in mouse. J. Physiol..

[14] Oke Y, Miwakeichi F, Oku Y, Hirrlinger J, Hulsmann S (2018). Cell type-dependent activation sequence during rhythmic bursting in the PreBotzinger complex in respiratory rhythmic slices from mice. Front. Physiol..

[15] Besser S (2015). A transgenic mouse line expressing the red fluorescent protein tdTomato in GABAergic neurons. PLoS ONE.

[16] Zeilhofer HU (2005). Glycinergic neurons expressing enhanced green fluorescent protein in bacterial artificial chromosome transgenic mice. J. Comp. Neurol..

[17] Kam K, Worrell JW, Janczewski WA, Cui Y, Feldman JL (2013). Distinct inspiratory rhythm and pattern generating mechanisms in the preBotzinger complex. J. Neurosci..

[18] Hintze JL, Nelson RD (1998). Violin plots: A box plot-density trace synergism. Am. Stat..

[19] Shao XM, Feldman JL (1997). Respiratory rhythm generation and synaptic inhibition of expiratory neurons in pre-Botzinger complex: Differential roles of glycinergic and GABAergic neural transmission. J. Neurophysiol..

[20] Baertsch NA, Baertsch HC, Ramirez JM (2018). The interdependence of excitation and inhibition for the control of dynamic breathing rhythms. Nat. Commun..

[21] Cui Y (2016). Defining preBotzinger complex rhythm- and pattern-generating neural microcircuits in vivo. Neuron.

[22] Janczewski WA, Tashima A, Hsu P, Cui Y, Feldman JL (2013). Role of inhibition in respiratory pattern generation. J. Neurosci..

[23] Marchenko V (2016). Perturbations of respiratory rhythm and pattern by disrupting synaptic inhibition within pre-Botzinger and Botzinger complexes. eNeuro.

[24] Ikeda K (2004). Malfunction of respiratory-related neuronal activity in Na+, K+-ATPase alpha2 subunit-deficient mice is attributable to abnormal Cl− homeostasis in brainstem neurons. J. Neurosci..

[25] Kuwana S, Okada Y, Sugawara Y, Tsunekawa N, Obata K (2003). Disturbance of neural respiratory control in neonatal mice lacking gaba synthesizing enzyme 67-kda isoform of glutamic acid decarboxylase. Neuroscience.

[26] Esclapez M, Tillakaratne NJK, Kaufman DL, Tobin AJ, Houser CR (1994). Comparative localization of two forms of glutamic acid decarboxylase and their mRNAs in rat brain supports the concept of functional differences between the forms. J. Neucosci..

[27] Richerson GB, Messer C (1995). Effect of composition of experimental solutions on neuronal survival during rat brain slicing. Exp. Neurol..

[28] Stosiek C, Garaschuk O, Holthoff K, Konnerth A (2003). In vivo two-photon calcium imaging of neuronal networks. Proc. Natl. Acad. Sci. USA.

[29] Schnell C, Fresemann J, Hulsmann S (2011). Determinants of functional coupling between astrocytes and respiratory neurons in the pre-Botzinger complex. PLoS ONE.

[30] Neher RA (2009). Blind source separation techniques for the decomposition of multiply labeled fluorescence images. Biophys. J..

